# Chromosomal localization of mutated genes in non-syndromic familial thyroid cancer

**DOI:** 10.3389/fonc.2024.1286426

**Published:** 2024-03-20

**Authors:** Yu-jia Jiang, Yun Xia, Zhuo-jun Han, Yi-xuan Hu, Tao Huang

**Affiliations:** ^1^ Department of Breast and Thyroid Surgery, Union Hospital, Tongji Medical College, Huazhong University of Science and Technology, Wuhan, China; ^2^ Hubei Bioinformatics and Molecular Imaging Key Laboratory, College of Life Science and Technology, Huazhong University of Science and Technology, Wuhan, China; ^3^ West China Biomedical Big Data Center, West China Hospital, Sichuan University, Chengdu, China

**Keywords:** thyroid carcinoma, familial non-medullary thyroid carcinoma, chromosomal locus, GWAS, WES, genetic mutation

## Abstract

Familial non-medullary thyroid carcinoma (FNMTC) is a type of thyroid cancer characterized by genetic susceptibility, representing approximately 5% of all non-medullary thyroid carcinomas. While some cases of FNMTC are associated with familial multi-organ tumor predisposition syndromes, the majority occur independently. The genetic mechanisms underlying non-syndromic FNMTC remain unclear. Initial studies utilized SNP linkage analysis to identify susceptibility loci, including the 1q21 locus, 2q21 locus, and 4q32 locus, among others. Subsequent research employed more advanced techniques such as Genome-wide Association Study and Whole Exome Sequencing, leading to the discovery of genes such as *IMMP2L*, *GALNTL4*, *WDR11-AS1*, *DUOX2*, *NOP53*, *MAP2K5*, and others. But FNMTC exhibits strong genetic heterogeneity, with each family having its own pathogenic genes. This is the first article to provide a chromosomal landscape map of susceptibility genes associated with non-syndromic FNMTC and analyze their potential associations. It also presents a detailed summary of variant loci, characteristics, research methodologies, and validation results from different countries.

## 1 Introduction

Familial non-medullary thyroid carcinoma (FNMTC) refers to a form of thyroid cancer that occurs in families, where there are at least two cases of thyroid cancer of follicular epithelial cell origin in first-degree relatives, excluding individuals with a history of exposure to known thyroid-cancer-causing factors (1, 2). It accounts for approximately 5% of all non-medullary thyroid carcinoma (1, 2). Epidemiological studies have shown that first-degree relatives of FNMTC patients have a 4 to 10 times higher risk of developing the disease compared to the general population. If two individuals in a family are affected, the probability of it being hereditary is estimated at 31% to 38%. If three or more individuals are affected, the probability of it being hereditary is over 94% (3).

Apart from a subset of FNMTC cases (approximately 5%) that are associated with syndromes FNMTC, the majority of FNMTC cases (approximately 95%) occur independently (4). This subset is also known as non-syndromic FNMTC, and the genetic mechanisms underlying this condition remain unclear. The initial studies utilized SNP linkage analysis to identify susceptibility genes for FNMTC, such as the 1q21 *fPTC/PRN* locus (5), 2q21 *NMTC1* locus (6), and 4q32 locus (7). Subsequently, the research progressed to using Genome-wide association study (GWAS), identifying genes like *IMMP2L* at 7q31.1 (8), *GALNTL4* at 11p15.4 (9), *WDR11-AS1* at 10q26.12 (10). Alternatively, Whole exome sequencing (WES) was employed to investigate FNMTC susceptibility genes by focusing on one or several pedigrees, resulting in the discovery of genes like *DUOX2* at 15q21.1 (11), *NOP53* at 19q13.33 (12), and *MAP2K5* at 15q23 (13).

However, it is important to note that the identified susceptibility genes, such as *MAP2K5* (15q23) (13–15), *HABP2* (10q25.3) (16–25), and the 19q13.2 locus (*TCO*) (26–30), primarily exist within specific families and may not be universally applicable across different families. FNMTC exhibits strong genetic heterogeneity, with each family having its specific pathogenic genes. It is the combination of these genes that may constitute the true susceptibility gene pool for FNMTC.

**Figure 1:**
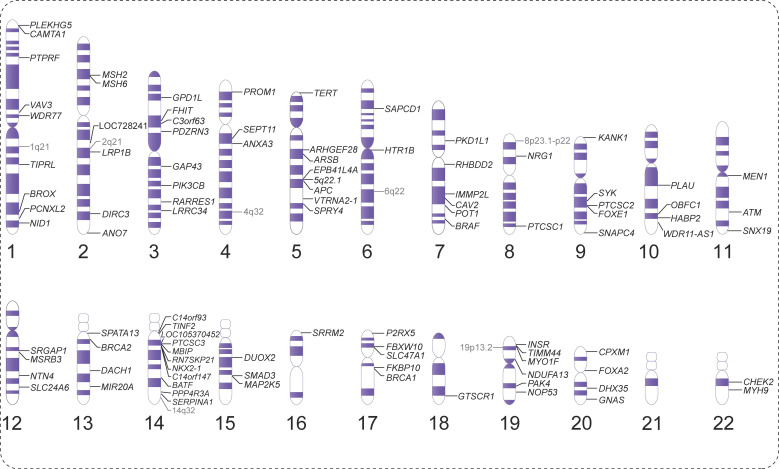
Overview of the chromosomal landscape of susceptibility genes associated with non-syndromic non-medullary thyroid carcinoma (NMTC).

Therefore, this article provides a comprehensive overview of the chromosomal landscape of susceptibility genes associated with non-syndromic FNMTC (**Figure 1**). Additionally, it offers a detailed summary of research findings, including information on variant loci, variant characteristics, research methodologies employed, and validation results from various countries.

## 2 Susceptibility chromosomal loci associated with risk of non-syndromic FNMTC

Initially, studies predominantly used SNP linkage analysis to identify susceptibility loci associated with FNMTC, but without specific genes. These loci are mostly related to specific types of NMTC. For instance, the discovery of the 1q21 locus is linked to papillary renal neoplasia (PRN) (31), the 2q21 locus is associated with follicular variant papillary thyroid carcinoma (fvPTC) (6), 4q32 locus was identified in 11 PTC patients and 2 families with undifferentiated thyroid cancer (7), 14q32 locus may be related to multinodular goiter (MNG) (32), and the 19p13.2 microdeletion may be associated with thyroid cancer with oncocytic (TCO) features (26), and so on. However, except for specific subtypes, the aforementioned susceptibility loci have been rarely validated in other NMTC (7, 26, 30, 30, 33, 34). For example, Suh (5) conducted a whole-genome SNP array analysis on 38 families. A significant correlation with chromosome locus 6q22 (the same chromosomal region as the PRN1 locus) and FNMTC was found, with an LOD score of 3.30. Similarly, Cavaco (33) performed SNP linkage analysis on 11 affected family members of Portuguese descent. The highest LOD score obtained was +4.41 at the FTEN locus on chromosome 8p23.1-p22. This suggests that the association between the chromosomal locus and FNMTC may not be universally applicable and could vary among different populations or families.

Significantly, the chromosomal locus at 19p13.2 has been implicated in numerous subsequent studies, exhibiting associations with various genes such as *INSR* (35), *TIMM44* (36), *NDUFA13/GRIM-19* (37) and *MYO1F* (38). Several studies have reported eosinophilic alterations in cells associated with TCO features (27–29). These alterations include abnormal mitochondrial proliferation in pro-oxidant or cancer cells. It is also the locus with the highest number of associated genes for FNMTC identified to date. This provides valuable insights for the fine mapping of susceptibility genes related to specific types of thyroid cancer. **Table 1** summarizes the chromosomal loci associated with FNMTC.

**Table 1:** Susceptibility chromosomal loci associated with risk of non-syndromic FNMTC.

Chromosomal loci	Tumor type	Population	Methods	Related to FNMTC	Ref.
1q21 (fPTC/PRN)	PTC and PRN	American	Linkage analysis	Yes	(5, 31)
2q21 (NMTC1)	PTC and fvPTC	Tasmanian	Linkage analysis	Yes	(6, 27, 28)
	NMTC and fvPTC	Greek and Portuguese	Linkage analysis	No	(30, 34)
4q32	PTC and ATC	American	Linkage analysis	Yes	(7)
	PTC	American and Polish	Linkage analysis	No	(7)
6q22	PTC	American	Linkage analysis	Yes	(5)
8p23.1-p22 (FTEN)	PTC	Portuguese	Linkage analysis	Yes	(33)
14q32 (MNG1)	PTC with MNG	Canadian	Linkage analysis	Yes	(32)
19p13.2 (TCO)	TCO, PTC and MNG	French, Canadian, Britisher, Italian, Sri Lankan, Maltese and Portuguese	Linkage analysis	Yes	(26–29)
	fvPTC	Greek	Linkage analysis	No	(30)

PTC, Papillary thyroid carcinoma; PRN, Papillary renal neoplasia; NMTC, Non-medullary thyroid carcinoma; ATC, Anaplastic thyroid carcinoma; MNG, Multinodular goiter; FvPTC, Follicular variant papillary thyroid carcinoma; TCO, Thyroid cancer with oncocytic.

## 3 RNA-associated susceptibility genes of FNMTC

RNA-associated susceptibility genes refer to genes that play important roles in the synthesis, modification, and regulation of RNA, and are associated with disease susceptibility. Currently, several RNA and genes associated with FNMTC have been identified, as shown in **Table 2**.

**Table 2:** RNA-associated susceptibility genes.

Chromosomal loci	Name	Type	Change	Tumor type	Population	Methods	Ref.
5q31.2	miR-886-3p (*VTRNA2-1*)	MicroRNA	Down-regulated	PTC	Chinese	Microarray analysis	(39)
13q31.3	miR-20a	MicroRNA	Down-regulated	PTC and ATC	Chinese	Microarray analysis	(40)
8q24	PTCSC1	LncRNA	Down-regulated	PTC and melanoma	American	Linkage analysis	(41)
9q22	PTCSC2	LncRNA	Down-regulated	PTC	American	3C assays	(42, 43)
14q13	PTCSC3	LincRNA	Down-regulated	PTC	American	Silico analysis	(44)
22q12.3	MYH9	Binding protein	Bind to PTCSC2	PTC	American	3C assays	(45)

Lnc-RNA, Long non-coding RNA; Linc-RNA, Long intergenic non-coding RNA; PTC, Papillary thyroid carcinoma; ATC, Anaplastic thyroid carcinoma; 3C assays, Chromosome conformation capture assays.

Firstly, the researchers found that miR-886-3p was down-regulated by 3-fold in FNMTC compared to SNMTC, while miR-20a was down-regulated by 4-fold in FNMTC compared to SNMTC. Overexpression of miR-886-3p was found to inhibit the expression of DNA replication gene *CDC6* and focal adhesion genes *PIP5K1C*, *PXN*, and *ZYX*. This overexpression of miR-886-3p resulted in the inhibition of cell proliferation and invasion, keeping the cells in the S phase. On the other hand, miR-20a is predominantly found in undifferentiated thyroid carcinoma and was shown to down-regulate the expression of LIMK1, a gene associated with reduced cell invasion (39, 40).

*Papillary thyroid carcinoma susceptibility candidate* (PTCSC) gene families belong to long non-coding RNA (lnc-RNA) that have been implicated in the development of papillary thyroid carcinoma (41). Currently, studies have shown that *PTCSC1*, *PTCSC2*, and *PTCSC3* are associated with the occurrence of FNMTC.

*PTCSC1*, also known as *AK023948*, is a gene that has been found to interact with DHX9 and the PI3K regulatory subunit p85-β (PIK3R2). The activation of AK023948 through this interaction leads to the phosphorylation and activation of protein kinase AKT. However, it is important to note that this specific association between AK023948 and breast cancer cell growth has not been explained or explored in the context of other studies on FNMTC (46).

An interaction between rs965513, a susceptibility SNP for FNMTC, *PTCSC2* has been observed. The SNP rs965513 in the 9q22 region play a crucial role in the genetic predisposition to PTC. And the [AA] risk genotype of rs965513 is associated with decreased expression of FOXE1, PTCSC2 unspliced transcript in unaffected thyroid tissue. The decrease in gene abundance is associated with dedifferentiation, which promotes malignant transformation (42, 43). Furthermore, MYH9 has been found to bind to the lncRNA PTCSC2, regulating the p53 signaling pathway by inhibiting FOXE1 expression (45).

*PTCSC3*, located downstream of SNP rs944289 at a distance of approximately 3.2 kb, exhibits characteristics of tumor suppressor genes (44). Rogounovitch et al. (47) confirmed the association of the T allele of SNP rs944289 with the risk of both PTC and benign thyroid nodules in the Japanese population.

## 4 Candidate genes identified by WES and susceptibility genes identified by GWAS

GWAS is a method used to detect genetic variations and polymorphisms across the whole genome in multiple individuals. GWAS aims to identify genetic variations that are most likely to influence a specific trait by examining the genotype-phenotype relationship. So far, GWAS studies from various populations have revealed several susceptibility genes for FNMTC research (8–10, 35, 48, 49) (**Tables 3**, **4**).

**Table 3:** Candidate genes identified by whole exome sequencing (WES) and susceptibility genes identified by genome-wide association studies (GWAS).

Tumor type	Population	Methods	Ref.	Chromosomal loci	Gene	AA Change
**PTC**	**Brazilian**	**WES**	(50)	4q21.21	*ANXA3*	D283N
				17q21.2	*FKBP10*	G188S
				12q22	*NTN4*	T157S
				17p13.2	*P2RX5*	L32Q
				6p21.33	*SAPCD1*	Q76T
				14q32.13	*SERPINA1*	G172W
				1p36.31	*PLEKHG5*	R937C
**PTC**	**Bedouin**	**WES**	(51)	5q13.2	*ARHGEF28*	N108S
				17p11.2	*FBXW10*	I440del
				17p11.2	*SLC47A1*	G288S
**NMTC**	**Chinese**	**Gene chip**	(52)	5q21	*APC*	L292F/A2778S
				7q34	*BRAF*	D22N
				2p16.3	*MSH6*	G355S/A36V
				2p21-p16	*MSH2*	L719F
				11q13.1	*MEN1*	G508D
				17q21.31	*BRCA1*	SS955S
				13q13.1	*BRCA2*	G2508S
				20q13.32	*GNAS*	Insertion
Tumor type	Population	Methods	Ref.	Chromosomal loci	Gene	SNP
**DTC**	**Italian**	**GWAS**	(8)	7q31.1	*IMMP2L*	rs10238549
						rs7800391
				3q25.32	*RARRES1*	rs7617304
				9q34.3	*SNAPC4*	rs10781500
				10q22.2	*PLAU*	rs2633322
				11q24.3-q25	*SNX19*	rs11823005
				18q22.2	*GTSCR1*	rs9951245
**DTC**	**Italian, Polish and Spanish**	**GWAS**	(48)	3p22.3	*GPD1L*	rs1159444
				1q24.2	*TIPRL*	rs2245026
				13q21.33	*DACH1*	rs2245026
				14q24.3	*BATF*	rs10136427
				20q11.23-q12	*DHX35*	rs7267944
				5q14.1	*ARSB*	rs13184587
**DTC**	**Italian**	**GWAS**	(9)	13q12.12	*SPATA13*	rs1220597
				20p11.21	*FOXA2*	rs1203952
				1p36.31-p36.23	*CAMTA1*	rs10864251
				2q14.3	*LOC728241*	rs1400967
				3p14.3	*C3orf63*	rs11130536
				3p13	*PDZRN3*	rs3863973
				9q22.2	*SYK*	rs290212
				14q13.1	*C14orf147*	rs4624074
**NMTC**	**Spanish and Southern European**	**GWAS**	(10)	10q26.12	*WDR11-AS1*	rs2997312
						rs10788123
						rs1254167
				6q14.1	*HTR1B*	rs4075570
**NMTC**	**European**	**GWAS**	(49)	1q42.2	*PCNXL2*	rs12129938
				3q26.2	*LRRC34*	rs6793295
				5q22.1	*NREP - EPB41L4A*	rs7322749
				10q24.33	*OBFC1*	rs7902587
				15q22.33	*SMAD3*	rs2289261
						rs56062135
**PTC**	**Korean**	**GWAS**	(35)	1p13.3	*VAV3*	rs4915076
				1q42.2	*PCNXL2*	rs4649295
				19p13.2	*INSR*	rs7248104
				12q14.3	*MSRB3*	rs11175834
				3p14.2	*FHIT*	rs9858271
				4q21.1	*SEPT11*	rs1874564
				12q24.13	*SLC24A6*	rs16934253
**PTC**	**Chinese**	**WES**	(53)	2q37.3	*ANO7*	rs57677160
				7q31.2	*CAV2*	rs8940
				9p24.3	*KANK1*	rs28374506
				3q22.3	*PIK3CB*	rs375961764
				7p12.3	*PKD1L1*	rs17131915
				1p34.2	*PTPRF*	rs17849118
				7q11.23	*RHBDD2*	rs11547498
**PTC**	**American,Icelander, and Britisher**	**GWAS-PRS**	(54)	1q42.2	*PCNXL2*	rs12129938
				2q35	*DIRC3*	rs11693806
				3q26.2	*LRRC34*	rs6793295
				5q22.1	*EPB41L4A*	rs73227498
				8p12	*NRG1*	rs2466076
				9q22.33	*FOXE1*	rs1588635
				10q24.33	*OBFC1*	rs7902587
				14q13.3	*LOC105370452*	rs368187
				14q13.3	*RN7SKP21*	rs116909374
				15q22.33	*SMAD3*	rs2289261
**STC**	**American**	**GWAS-PRS**	(55)	1q42.2	*PCNXL2*	rs12129938
				2q35	*DIRC3*	rs11693806
				3q26.2	*LRRC34*	rs6793295
				5p15.33	*TERT*	rs10069690
				5q22.1	*EPB41L4A*	rs73227498
				8p12	*NRG1*	rs2466076
				9q22.33	*FOXE1*	rs1588635
				10q24.33	*OBFC1*	rs7902587
				14q13.2	*LOC105370452*	rs368187
				14q13.3	*RN7SKP21*	rs116909374
				15q22.33	*SMAD3*	rs2289261
						rs56062135
**DTC**	**Korean**	**GWAS-PRS**	(56)	2q35	*DIRC3*	rs6759952
				3q13.31	*GAP43*	rs13059137
				4p15.32	*NRG1*	rs7834206
				2q22	*PROM1_*	rs72616195
				12q14.3	*LRP1B_*	rs1369535
				12q14.3	*MSRB3-AS1*	rs11175834
**PTC**	**European**	**FCVPPv2**		20p13	*CPXM1*	rs145736623

PTC, Papillary thyroid carcinoma; NMTC, Non-medullary thyroid carcinoma; DTC, Differentiated thyroid carcinoma; STC, Subsequent thyroid cancer; WES, Whole exome sequencing; GWAS, Genome-wide association study; PRS, Polygenic risk score; FCVPPv2, Familial Cancer Variant Prioritization Pipeline version 2.

**Table 4:** Identified susceptibility genes associated with Familial non-medullary thyroid carcinoma.

Chromosomal loci	Gene	SNP	Tumor type	Population	Methods	Related to FNMTC	Ref.
**2q35**	***DIRC3***	rs966423	Thyroid cancer with low TSH levels, PTC	Icelander, Spanish, American, Netherlander, Korean, Chinese and Polish	Linked analysis, GWAS	Yes	(35, 57–60)
		rs966423	PTC	European	GWAS	NO	(10)
		rs12990503	DTC	Korean	GWAS	Yes	(35)
		rs16857609	DTC	European	GWAS	Yes	(61)
		rs6759952	DTC	Italian, Korean	GWAS	Yes	(8, 35)
		rs11693806	NMTC	Europeans	GWAS	Yes	(49)
**5p15.33**	***TERT***	NA	NMTC	Portuguese	Targeted DNA sequencing	Yes	(62)
		rs2736100	Thyroid cancer	European, Chinese	Linked analysis, GWAS	Yes	(49, 63)
		rs10069690	Thyroid cancer	European	GWAS	Yes	(49)
**8p12**	***NRG1***	rs6996585	DTC	Korean	GWAS	Yes	(35)
		rs12542743	DTC	Korean	GWAS	Yes	(35)
		rs2439302	Thyroid cancer with low TSH levels, PTC	Icelander, Chinese, Japanese, Kazakh, American and Polish	Linked analysis, GWAS	Yes	(47, 57–60)
		rs2466076	NMTC	Icelander	GWAS	Yes	(49)
		rs2439304	DTC	European, Melanesian and Polynesian	GWAS	Yes	(61)
**9q22**	***FOXE1/*** ***TTF-2***	rs1867277	PTC	Spanish, Italian and Portuguese	SNP Genotyping, Targeted DNA sequencing	Yes	(64–66)
		rs1867278	PTC	Portuguese	Targeted DNA sequencing	Yes	(65, 67)
		rs1867279	PTC	Portuguese	Targeted DNA sequencing	Yes	(65)
		rs1867280	PTC	Portuguese	Targeted DNA sequencing	Yes	(65)
		rs3021523	PTC	Portuguese	Targeted DNA sequencing	Yes	(65)
		rs7849497	PTC	Portuguese	Targeted DNA sequencing	Yes	(65)
		rs7037324	NMTC	Spanish and Southern European	GWAS	Yes	(10)
		rs7028661	NMTC	Korean	GWAS	Yes	(10, 35)
		rs1588635	NMTC	Korean	GWAS	Yes	(35)
		rs10122541	NMTC	Spanish and Southern European	GWAS	Yes	(10)
		rs3021526	NMTC	Portuguese	Targeted DNA sequencing	Yes	(65)
		PolyAla	NMTC	Portuguese	Targeted DNA sequencing	Yes	(65)
		rs965513	PTC, FTC	Icelander, Columbus, Spanish, Portuguese, Korean, Japanese, Chinese and Belarusian	GWAS, SNP Genotyping, Targeted DNA sequencing	Yes	(35, 47, 58, 65, 66, 68–70)
		rs10759944	PTC	NA	SNP Genotyping	Yes	(66)
**10q25.3**	***HABP2***	rs2286742	PTC	NA	Targeted next-generation sequencing	Yes	(71)
		rs3740530	PTC	NA	Targeted next-generation sequencing	Yes	(71)
**11q22.3**	***ATM***	rs1801516	PTC	Polish	SNP Genotyping	NO	(72)
		rs373759	Sporadic PTC	Asian	SNP Genotyping	Yes	(73)
		rs664143 rs4585	Sporadic PTC	Korean	SNP Genotyping	Yes	(74)
**12q14**	***SRGAP1***	rs781626187 rs797044990 rs114817817	PTC	American	linked analysis	Yes	(75)
			PTC	American and Poland	linked analysis	No	(75)
		rs2168411	PTC	American and Poland	linked analysis	Yes	(75)
**14q13**	***NKX2-1/TTF-1***	rs34081947 rs368187	NMTC	European	GWAS	Yes	(49)
		rs944289	PTC and MNG	Chinese, Japanese, Korean, Spanish and Southern European	GWAS	Yes	(10, 35, 58, 69)
		rs944289	Exposed to radiation	Belarusian	GWAS	No	(70)
**14q13.3**	***MBIP***	rs116909374	NMTC	Icelander	GWAS	Yes	(49, 57)
		rs34081947 rs368187	DTC	Korean	GWAS	Yes	(35)
**17q21.31**	***BRCA1***	rs16941	PTC	Polish	SNP Genotyping	Yes	(72)
Chromosomal loci	Gene	AA change/Base change	Tumor type	Population	Methods	Related to FNMTC	Ref.
**1p13.2**	***WDR77***	R198H	NMTC	Chinese	WES	Yes	(76)
		c.619 + 1G>C				Yes	
**1q41**	***BROX***	R40fs	NMTC	Italian	WES	Yes	(77)
		c.-2898C > T				Yes	
**1q42.3**	***NID1***	I657M	PTC	Brazilian	WES	Yes	(78)
**5q31.3**	***SPRY4***	T234M	PTC	Portuguese	WES	Yes	(79)
**7q31.33**	***POT1***	K90E, I78T, E344*, D598Sfs*22r.125_255del	Familial melanoma with thyroid cancer	American	WES	Yes	(80)
		V29L	PTC	Italian	WGS	Yes	(81)
		V29L	PTC	Spanish	WES	NO	(82)
**10q25.3**	***HABP2***	G534E	PTC	American	WES	Yes	(16)
		G534E	PTC	Australian, Polish, Brazilian, Italian, middle eastern, Britisher, European, American, etc	Sanger sequencing and WES	No	(17–24)
		R122W	PTC	European	Sanger sequencing	Yes	(24)
		R122W	NMTC	Italian	Targeted DNA sequencing	No	(25)
**14q11.2**	***C14orf93/RTFC***	V205M	PTC	Chinese	WES and parallel linkage analysis	Yes	(83)
**14q12**	***TINF2***	W198fs	PTC and PTC with melanoma	American	WES, Sanger sequencing	Yes	(84)
		D123N	PTC	American	WES and WGS	Yes	(84, 85)
**14q13**	***NKX2-1/TTF-1***	A339V	PTC with MNG	Chinese	Sanger sequence	Yes	(86)
**14q32**	***PPP4R3A***	D409N	PTC	Chinese	WES	Yes	(87)
**15q21.1**	***DUOX2***	Y1203H	PTC	American	WES	Yes	(11)
**15q23**	***MAP2K5***	A321T/M367T	PTC	Chinese	WES	Yes	(13)
		c.G961A, c.T1100C	NMTC	Italian	Sanger sequencing	No	(14, 15)
**16p13.3**	***SRRM2***	S346F	PTC	American	WES	Yes	(88)
**17q21.31**	***BRCA1***	SS955S	PTC	Chinese	Gene Chip	Yes	(52)
**19p13.2**	***GRIM-19/NDUFA13***	C77T, A26V G264C, K88N, A247G, S83G, G593C, R198P	Hürthle cell tumors	Portuguese	Targeted DNA sequencing	Yes	(37)
**19p13.2**	***TIMM44***	C925A, P308Q, G1274A, Silent	Thyroid cancer with *TCO*	Austrian	Targeted DNA sequencing and Microsatellite analysis	Yes	(36)
**19p13.2**	***MYO1F***	G134S	Thyroid cancer with *TCO*	Lyons	WES	Yes	(38)
**19q13.2**	***PAK4***	I417T	PTC	Chinese	WES	Yes	(89)
**19q13.33**	***NOP53***	D31H	PTC	Spain	WES and Sanger sequencing	Yes	(12)
**22q12.1**	***CHEK2***	1100delC, IVS21G>A, del5395, I157T	PTC	Polish	SNP Genotyping and Sanger sequencing	Yes	(72, 90)
		Y139X	PTC	Chinese	WGS	Yes	(91)

AA, Amino acids; NA, Not available; FNMTC, Familial non-medullary thyroid carcinoma; PTC, Papillary thyroid carcinoma; NMTC, Non-medullary thyroid carcinoma; TSH, Thyroid stimulating hormone; FTC, Follicular thyroid carcinoma; DTC, Differentiated thyroid carcinoma; MNG, Multinodular goiter; WES, Whole exome sequencing; GWAS, Genome-wide association study; WGS, Whole genome sequencing.

Although GWAS studies have identified numerous risk SNPs, FNMTC cannot currently be explained by single-gene variants. Therefore, researchers have started using polygenic risk scores (PRS) to assess the risk of developing thyroid cancer.

For example, a study utilizing GWAS data from the United States, Iceland, and the United Kingdom proposed a 10-SNP PRS assessment model (rs12129938, rs11693806, rs6793295, rs73227498, rs2466076, rs1588635, rs7902587, rs368187, rs116909374, and rs2289261), which could be applied for personalized assessment of thyroid cancer susceptibility (54). A study analyzed the PRS of 12 thyroid cancer-associated SNPs (rs11693806, rs2466076, rs1588635, rs368187, rs116909374, rs12129938, rs6793295, rs73227498, rs7902587, rs2289261, and rs56062135) in childhood cancer survivors of European population. The findings showed that for each one standard deviation increase in PRS (1.57) (55). Furthermore, another study compared the high PRS tertile with the low PRS tertile and found that the PRS of six SNPs (rs6759952, rs13059137, rs7834206, rs72616195, rs1369535, rs11175834) increased the risk of thyroid cancer by 3.9 times (56). These studies suggest that utilizing PRS can better assess an individual’s risk of developing thyroid cancer. However, it is important to note that PRS is still evolving, and further validation and refinement are necessary before widespread clinical implementation. (**Table 3**).

In addition to the aforementioned PRS method, Asta et al. (92) introduced the familial cancer variant prioritization pipeline (FCVPP), which is a comprehensive approach for analyzing germline genomes in Mendelian cancer pedigrees. This pipeline involves variant calling, quality control, frequency screening, segregation analysis, and bioinformatics assessment. By considering these steps and ensuring accurate pedigree information, researchers can enhance the likelihood of identifying novel genes associated with Mendelian types of cancer. Building upon this, Abhishek et al. (93) introduced FCVPPv2, an enhanced pipeline for prioritizing variants in pedigrees that incorporates multiple tools to assess variant deleteriousness and intolerance scores. The pipeline also encompasses the evaluation of non-coding regions using various datasets and tools. Application of the pipeline on a papillary thyroid cancer family revealed one variant in the *CPXM1* gene (c.G1717A:p.G573R), suggesting its potential involvement in tumorigenesis (**Table 3**). However, further functional characterization is required to validate its role in cancer predisposition. Overall, FCVPPv2 provides a comprehensive approach for predicting high-risk cancer predisposing variants in familial cancer pedigrees.

WES is also widely used for the screening of candidate genes. However, most of these loci have not been mentioned or functionally validated in other studies, as indicated in **Table 3**.

For example, Yang et al. (52), identified 10 germline mutations were found in 8 genes, including *APC* (L292F/A2778S), *BRAF* (D22N), *MSH6* (G355S/A36V), *MSH2* (L719F), *MEN1* (G508D), *BRCA1* (SS955S), *BRCA2* (G2508S), and *GNAS* insertions. The mutation rate of these eight genes was found to be 53.2% in the FNMTC group and 31.3% in the SNMTC group. However, it is important to note that the sample size might have contributed to the lack of statistical significance in distinguishing FNMTC from SNMTC, with an odds ratio of 2.46 and a P-value of 0.16. Furthermore, Majdalani et al. (51) suggested that *ARHGEF28* is a promising candidate due to its high expression in the thyroid and potential associations with other genes. Protein-protein interactions suggest that ARHGEF28 may predispose individuals to PTC through associations with SQSTM1-TP53 or PTCSC2-FOXE1-TP53. Zhu et al. (53) identified Seven novel candidate FNMTC pathogenic genes (*ANO7*, *CAV2*, *KANK1*, *PIK3CB*, *PKD1L1*, *PTPRF*, and *RHBDD2*). Notably, three of these genes (*PIK3CB*, *CAV2*, and *KANK1*) have been linked to tumorigenesis through the PI3K/Akt signaling pathway. Sarquis et al. (50) performed WES on three FNMTC pedigrees in Brazil and identified seven potential FNMTC susceptibility genes. The three FNMTC pedigrees had a total of 11 patients with an average age of diagnosis at 41.5 years.

Although this class only involves the analysis of candidate genes at the level of bioinformatics, it may also have certain significance for expanding the FNMTC gene library. In the future, more verification is needed to confirm the contribution of these genes.

## 5 Identified susceptibility genes associated with FNMTC

In the FNMTC susceptibility gene map, most of the genes have been validated through other pedigrees or functional studies, but there is controversy surrounding many genes. Some of them may be susceptibility genes for specific subtypes of NMTC, while others may only belong to specific certain populations. This once again highlights that the genetic susceptibility of FNMTC is not determined by a single gene. Therefore, the following text and **Table 4** provide detailed information.

### 5.1 1p13.2 *WDR77*

WD repeat domain 77 (WDR77), also known as methyltransferase-like protein 50 and androgen receptor-associated protein p44, was studied by Zhao et al. through WES in two unrelated Chinese pedigrees with FNMTC. In one pedigree, a missense mutation (p.R198H) was found in exon 6 of *WDR77*, while in the other pedigree, a splice site mutation (c.619 + 1G>c) was identified at the 5’ end of intron 6 (76).

WDR77 forms a complex with protein arginine methyltransferase 5 (PRMT5) to regulate the formation of histone H4 arginine 3 dimethylation (H4R3me2) in cells. The functional activity of WDR77 protein is dependent on its subcellular localization. For example, in early developing prostate epithelial cells, WDR77 is located in the cytoplasm and plays a crucial role in cell proliferation (94, 95). Functional studies have shown that mutations in WDR77 disrupt the formation of the PRMT5/WDR77 complex, resulting in reduced H4R3me2 levels within cells. Knockdown of the WDR77 gene leads to an increased proliferation rate in thyroid cancer cells (76).

### 5.2 q41 BROX

Pasquali et al. conducted WES analysis on five FNMTC pedigrees and identified two pedigrees with loss-of-function mutations in the *BROX* gene. In one pedigree, a frameshift deletion mutation (chr1:222892283 NM_001288579:c.119delG:p.Arg40fs) occurred in the *BROX* gene, while in another pedigree, a missense mutation (chr1:222886144 NM_144695:c.2898C>T) was observed (77). However, there is currently no direct evidence of a functional effect associated with the second variant.

BROX encodes human Brox, which contains a Bro1 domain-like sequence involved in the endosomal sorting of cargo proteins and degradation processes in lysosomes, such as integrin and EGFR (96–98). The researchers hypothesized that a *BROX* mutation is sufficient to alter the degradation of EGFR, leading to the accumulation of EGFR and subsequently abnormal cell growth (77).

### 5.3 1q42.3 *NID1*

WES was performed on a Brazilian family with a history of PTC. Previously reported FNMTC susceptibility genes were examined, and novel candidate genes were identified using PhenoDB. No variants in known FNMTC susceptibility genes co-segregated with the disease. However, a missense variant (c.1971T>G:p.Ile657Met) in the *NID1* gene co-segregated with the disease and had a low allele frequency. In silico analysis suggested its deleteriousness, and NID1 expression was observed in PTC cells but not normal thyroid tissue. TCGA data showed higher NID1 expression associated with the risk of recurrence and lateral neck lymph node metastasis in PTCs. The *NID1* variant in this study shows potential as a novel FNMTC predisposing gene (78).

### 5.4 2q35 *DIRC3*

*DIRC3* (*Disrupted in Renal Cancer 3*) was already known to associate hereditary renal cancer and was believed to have tumor suppressor activity (99). In a GWAS study involving 561 Icelandic thyroid cancer cases and 40,013 controls, rs966423 variant of the *DIRC*3 was found to be associated with thyroid cancer risk and levels of thyroid-stimulating hormone for the first time (57). Multiple reports have shown the prognostic significance of the rs966423 variant and its pathogenic effects in cases of DTC (35, 57–60). Subsequent replication studies identified additional variants, rs6759952 (8, 35) and rs12990503 (35). However, Mankickova et al. (10) were unable to establish an association between rs966423 and thyroid cancer in European populations. More recently, GWAS analysis discovered novel variants, 11693806 (49) located near *DIRC3*, and rs16857609 (61), that are associated with European ancestry.

### 5.5 5p15.33 *TERT*

Ge et al. (63) conducted a study on thyroid cancer in Chinese populations. They found that *TERT* rs2736100 was significantly associated with thyroid cancer in the Chinese cohort. Other study in populations of European ancestry confirmed this association (rs2736100). Additionally, they identified rs10069690 as the variant with the strongest association in this region and suggested that these two variants likely represent the same underlying association signal, with rs10069690 providing a slight refinement in the European ancestry study groups compared to the Chinese population (49).

In an article that examined 75 FNMTC families, the promoter region of the *TERT* gene was sequenced. Somatic mutations in the *TERT* promoter, as well as *RAS* and *BRAF* mutations, were evaluated, along with the entire *EIF1AX* gene in 54 familial cases of thyroid tumors. The study found that FNMTC is associated with short telomeres, an increased copy number of the *TERT* gene, and elevated telomerase activity in both germline and somatic cells.

Additionally, the study suggested that *TERT* promoter and *EIF1AX* mutations are infrequently involved in the development of FNMTC. However, *TERT* mutations were more frequently observed in late-onset FNMTC cases compared to *BRAF* mutations, indicating a potential correlation between TERT mutations and the late onset of FNMTC (62).

### 5.6 5q31.3 *SPRY4*

Marques et al. (79) performed WES on leukocyte DNA from six affected members of FNMTC family. Shared genetic variants were identified through bioinformatic analysis and validated using Sanger sequencing. After filtering, a specific variant (c.701C>T, p.Thr234Met) in the *SPRY4* gene was identified as the most promising. *In vitro* experiments showed that this variant increased cell viability and colony formation, indicating enhanced proliferation and clonogenic capacity. Analysis suggested that the *SPRY4* variant acted through the mitogen-activated protein kinase/extracellular signal-regulated kinase pathway, with increased sensitivity to a MEK inhibitor in thyroid cancer cells.

### 5.7 7q31.33 *POT1*

*POT1*, also known as *GLM9*, *CMM10*, and *HPOT1*, is involved in telomere maintenance. Mutations in *POT1* were first reported in familial melanoma, where some family members also developed thyroid cancer (80, 100). Capezzone et al. found that peripheral blood samples from familial PTC patients exhibited an imbalance in the telomere-telomerase complex, characterized by shortened telomeres and *TERT* gene amplification (101). Richard et al. used functional variant approaches to confirm the association between low-frequency intronic regulatory *POT1* variants in survivors of childhood cancer and subsequent development of malignant thyroid tumors. Intronic variations in *POT1* may affect key proteins involved in telomere maintenance and genomic integrity (102).

Recently, Srivastava et al. (81) reported a pedigree with FNMTC, in which a novel mutation (p.Val29Leu) was identified in the *POT1* gene among family members. *In vitro* experiments showed that the p.Val29Leu mutation in the *POT1* gene exhibited increased telomere length, indicating loss of function caused by the mutation and resulting in telomere dysfunction. However, Orois et al. (82) did not find potential pathogenic mutations in the *POT1* gene through WES in four FNMTC pedigrees from Spain.

### 5.8 8p12 *NRG1*

The NRG1 gene encodes neuregulin 1, a signaling protein involved in the development of both malignant and benign thyroid tumors. Additionally, it plays a role in protecting against oxidative damage, and influencing cell proliferation (57, 59, 103, 104).

Studies identified strong associations in the *NRG1* gene, specifically with rs6996585, rs12542743 and rs2439302 (35, 47, 57–60). Guibon et al. (61) conducted fine mapping of the 8p12 (*NRG1*) locus, identifying rs2439304 as being associated with DTC. Furthermore, another variant at the *NRG1* locus (rs2466076) was found to be associated with 3001 NMTC cases and 287,550 controls from Iceland (49).

### 5.9 9q22 FOXE1/TTF-2

The *FOXE1* gene, located on chromosome 9q22.33, encodes the *FOXE1 transcription factor* (also known as *thyroid transcription factor 2*, *TTF-2*). This gene is involved in regulating the expression of genes such as thyroglobulin and thyroid peroxidase, and it plays an essential role in the formation and development of the thyroid gland.

Regarding the association of *FOXE1* with FNMTC, several studies have been conducted to investigate its potential role. In one study (64), the SNP rs1867277 variation at the 5’UTR was found to regulate *FOXE1* transcription. Another study (65, 67) tested the *FOXE1* gene in 60 Portuguese FNMTC families and 80 SNMTC cases and identified ten germline variants in the promoter and coding sequence of the gene. One variant, c.743C > G (p.A248G), showed gene-disease co-segregation in one FNMTC family and was detected in a disseminated PTC case.

In another study (66), a total of 672 patients from 133 FNMTC progeny were tested at 11 known candidate loci, including the chromosomal locus 9q22.33 near *FOXE1*. The SNPs rs965513 and rs10759944 at 9q22.33 showed the most consistent association with FNMTC. However, Bonora et al. (66) reported that among the previously mentioned rs944289 (68) and rs1867277 (64) SNPs, only rs1867277 was consistently associated with FNMTC. Furthermore, validation from 95 FNMTC cases carrying three risk alleles of related SNPs (rs965513, rs10759944, rs1867277) at the 9q22.33 locus did not find any deleterious missense mutations in the entire coding sequence of *FOXE1*. Therefore, the aforementioned *FOXE1*-associated SNPs were not consistently shown to be associated with FNMTC.

A study conducted on the Icelandic population and people of European ancestry (67) showed that two simultaneous polymorphic changes (rs944289 and rs965513) near the *FOXE1* gene increased the risk of PTC and follicular thyroid carcinoma (FTC) development. Overall, it should be noted that not all literature supports a direct association between the *FOXE1* gene and FNMTC.

### 5.10 10q25.3 *HABP2*

Gara et al. (16) performed exome sequencing on seven FNMTC patients from one family, in which the HABP2 G534E mutation was found to promote tumorigenesis, growth, invasion, and loss of tumor suppression. Weeks et al. (17) conducted Sanger sequencing on 37 Australian families with FNMTC to validate the presence of the HABP2 G534E mutation. Additionally, WES was performed on 59 participants from 20 families to validate the *HABP2* mutation. However, no explainable HABP2 G534E mutation was detected in the Australian family. Furthermore, studies conducted in regions including Europe and the Middle East have also failed to replicate this data (17–24).

In another study by Kern et al. (24), Sanger sequencing was performed on 20 patients with familial PTC from 11 European families. They identified c.1601G > A (p.Gly534Glu) mutations in two patients at the heterozygous level and c.364C > T (p.Arg122Trp) mutations in three patients. The minor allele frequencies (MAF) for these mutations were 5.0% and 7.5%, respectively. Therefore, these findings do not support the pathogenicity of the *HABP2* c.1601G > A (p.Gly534Glu) variant. However, the study highlighted the presence of a novel mutation, c.364C > T (p.Arg122Trp), which was not found in another study (25) involving 32 Italian FNMTC families that did not identify the HABP2 R122W variant.

Finally, additional *HABP2* variants (rs2286742 and rs3740530) were identified, which have the potential to increase the risk of PTC in a recessive model, respectively (71).

Therefore, considering potential ethnic variations across different regions, further investigation is warranted in a broader cohort of patients with familial PTC to thoroughly evaluate its pathogenicity.

### 5.11 11q22.3*ATM* 17q21.31 *BRCA1* 22q12.1 *CHEK2*

Ionizing radiation has been documented to directly increase the risk of thyroid and breast cancer by causing DNA double-strand breaks, thereby promoting carcinogenesis. The process of DNA damage repair is influenced by genetic polymorphisms and mutations in the ATM-BRCA1-CHEK2 pathway. In mammalian cells, DNA double-strand breaks activate the ATM kinase, which phosphorylates and activates CHEK2. This activation leads to the phosphorylation of BRCA1, initiating DNA repair. If the repair fails, apoptosis may occur (72).

Wojcicka et al. conducted a study and found that certain genetic variations, such as ATM D1853N, BRCA1 E1038G, and CHEK2 I157T, were associated with an increased susceptibility to PTC. They also discovered that specific single-nucleotide polymorphisms (SNPs), namely rs17879961 in *CHEK2* and rs16941 in *BRCA1*, were significantly associated with PTC susceptibility. However, they did not find an association between *ATM* rs1801516 and PTC (72).

In another study, it was suggested that polymorphic changes in the *ATM* gene increased the risk of sporadic PTC and DTC in Caucasians (105, 106). Gu et al. (73) investigated the relationship between polymorphic changes in *ATM* and the increased risk of PTC in an Asian population. Among the four studied polymorphisms (rs664677, rs373759, rs4988099, rs189037), only rs373759 was found to be associated with an increased risk of PTC development. Strong linkage disequilibrium was observed among three *ATM* SNPs (rs373759, rs664143, and rs4585). The presence of the *ATM* haplotype (C-G-T) +/- was associated with a lower risk of PTC compared to the absence of this haplotype (C-G-T) -/-, suggesting the potential role of *ATM* genetic polymorphisms in thyroid cancer development in the Korean population (74).

Siołek et al. (90) demonstrated a significant correlation between carrying *CHEK2* mutations (such as 1100delC, IVS21G > A, del5395, and I157T) and an increased risk of PTC. To further investigate this relationship, they genotyped 468 PTC patients and 468 age- and sex-matched cancer-free controls. Among the PTC cases, 73 out of 468 (15.6%) carried *CHEK2* mutations, while only 28 out of 460 (6.0%) of the controls had these mutations. Zhao et al. (91) conducted a study involving whole genome sequencing of from two PTC patients in the same family. As a result, a new heterozygous germline mutation in *CHEK2* (c.417C→A) was identified in all affected members of the family.

Functional analysis revealed that the *CHEK2* c.417C > A variant introduces a premature termination codon (Y139X), resulting in the production of a truncated protein. This loss-of-function variant led to reduced p53 phosphorylation and decreased abundance of the p53 protein. In addition to the Y139X variant, two rare missense variants (R180C and H371Y) were also detected in the *CHEK2* gene.

### 5.12 12q14 SRGAP1

An SNP-linked analysis of 38 families suggested that three variants (rs781626187, rs797044990 and rs114817817) in the *SRGAP1* gene might serve as potential candidate genes for FNMTC (75). These variants could potentially lead to the loss of gene function, resulting in the inactivation of the CDC42 protein. This, in turn, can impact the regulation of intracellular signaling networks, influencing various signaling pathways and promoting tumorigenesis (107). Additionally, the identified variants were not found in population studies conducted in Ohio or Poland. However, a different SNP (rs2168411) of the *SRGAP1* gene demonstrated an association with PTC in both the Ohio and Poland populations (75).

### 5.13 14q11.2 C14orf93/RTFC

Liu et al. (83) conducted a study involving one Chinese family with FNMTC using WES and linkage analysis and validated their findings in 14 additional FNMTC families. Through this analysis, they identified six candidate genes: *C14orf93/RTFC* (chr14:23465462), *PYGL* (chr14:51383415), *BMP4* (chr14:54418885), *PLCB4* (chr20: 9440331), *CCDC54* (chr3:107097080), and *EIF2AK4* (chr15:40268998). Further screening based on allele frequency and linkage analysis revealed that *C14orf93/RTFC* was the only remaining candidate gene among the six identified. The other five genes were excluded from further consideration. In particular, the V205M *RTFC* mutant was found to be the only oncogenic mutation that promoted cell survival, cell migration, and colony-forming capacity under starvation conditions.

### 5.14 14q12 *TINF2*

The study involved genome sequencing analysis of a large family with cases of PTC and melanoma. Screening for coding variants in the shelterin genes was conducted in 24 families using WES. A frameshift mutation in the *TINF2* gene (TINF2 p.Trp198fs) was identified, showing complete co-segregation with PTC and melanoma in the key family. This mutation was unique to this family. Reduced TINF2 expression and disrupted binding to TERF1 were observed in individuals with the mutation. These individuals also exhibited significantly longer telomeres compared to unaffected family members and healthy controls. In addition, rare missense and synonymous variants in *TINF2* and *ACD* were found in some families (84, 85).

### 5.15 14q13 NKX2-1/TTF-1,MBIP

The *TTF-1* gene is located on chromosome 14q13 and encodes the *thyroid transcription factor-1* (*TTF-1)*. This protein plays a role in activating the transcription of thyroglobulin, thyroid peroxidase, and thyroid stimulating hormone receptors.

Ngan et al. (86) conducted targeted DNA sequencing of the *SRGAP1* gene in 20 patients with PTC who had a history of MNG, as well as in 284 patients with PTC without a history of MNG. Among the latter group, four patients with PTC were found to have the *NKX2-1/TTF-1* mutation pattern.

Functional experiments have indicated that mutations in this gene, such as the TTF-1 A339V mutation, can stimulate thyroid cell proliferation independently of thyroid-stimulating hormone. These mutations have also been shown to activate STAT3 and Akt signaling pathways and increase the expression of cyclin D2. These findings suggest that the TTF-1 A339V mutation may be associated with the development of PTC. However, these results have not been consistently confirmed in other experiments (108).

Gudmundsson et al. (47, 68) discovered that downstream of the *NKX2-1* gene, there is a risk allele T for the SNP rs944289. Liao et al. (109) conducted genetic testing on a Chinese family and found that the T allele of rs944289 is a risk allele for members of this family to develop MNG and PTC. This association was also confirmed by other countries (10, 35, 58, 69). Another genome-wide study conducted by Takahashi et al. (70) focused on Belarusians who were exposed to radiation from the Chernobyl incident at 18 years of age or younger. However, this study did not find an association between radiation-associated PTC and the SNP rs944289 located at the 14q13.3 locus. Other relevant variants, such as rs34081947 and rs368187, were initially identified in a GWAS conducted on European populations (49).

In addition to rs944289, Gudmundsson also proposed that the variants (rs116909374 on 14q13.3) were significantly associated with thyroid cancer. On chromosome 14q13.3, the nearby gene *MBIP* and *NKX2-1/TTF-1* are important candidates to consider for their roles in thyroid development (57).

### 5.16 15q21.1 *DUOX2*

Ohye et al. (11) utilized WES to identify a novel germline mutation in the *DUOX2* gene associated with FNMTC. This mutation, known as DUOX2 Y1203H, affects the function of *DUOX2*, which is responsible for hydrogen peroxide (H2O2) production in the thyroid gland. H2O2 is essential for iodide tyrosine residue oxidation and the synthesis of thyroxine and triiodothyronine by thyroid peroxidase.

The study found that DUOX2 Y1203H is a functionally active mutation, and it was observed to be highly expressed in normal thyroid tissue, potentially increasing the risk of tumorigenesis. Notably, the expression of DUOX2 was found to be elevated in patients harboring the rs965513 variant, suggesting a potential link between dysregulated H2O2 metabolism mediated by *DUOX2* and hereditary thyroid carcinoma with either high or low penetrance. However, a study on 33 Italian FNMTC pedigrees did not find these mutations (110).

### 5.17 15q23 MAP2K5

Ye et al. (13) conducted a study utilizing WES in 33 patients with FNMTC and further validated their results in an additional 44 intra-family patients. Through this analysis, they identified five candidate genes: *MAP2K5*, *ZNF500*, *MUC6*, *IGSF3*, and *FRG1*.

An interesting aspect to consider is that the pathogenesis of FNMTC diverges from the classical MAP2K1/2-ERK1/2 signaling pathway typically associated with sporadic cases. In FNMTC, there is a distinct activation of the MAP2K5-ERK5 pathway, which subsequently causes changes in gene expression downstream. These alterations ultimately drive the malignant transformation of thyroid epithelial cells.

Additionally, a separate study targeting *MAP2K5* DNA assays in Italian families did not yield any positive results (14, 15).

### 5.18 16p13.3 *SRRM2*

This study (88) involved a sample of 6 FNMTC patients from a single family, which was then validated by including an additional 138 FNMTC families. The study also screened for the presence of the FNMTC susceptibility gene *SRRM2* in 1170 sporadic PTC cases from Ohio, as well as 1404 healthy controls.

The data obtained from this study suggest that the S346F mutation in the *SRRM2* gene may play a role in PTC development. This mutation affects the splicing process and leads to variable splicing of downstream target genes. A total of 7 *SRRM2* mutations were identified in 7 PTC cases, while no mutations were found in the 1404 controls. However, it is worth noting that these mutations were not detected in the 138 FNMTC families analyzed.

### 5.19 19p13.2 GRIM-19/NDUFA13

There is a cluster of genes involved in mitochondrial metabolism, including *GRIM-19*. In a study of 52 thyroid tumors, researchers searched for *GRIM-19* mutations. They found somatic missense mutations in three sporadic Hurthle cell carcinomas and a germline mutation in a Hurthle cell papillary carcinoma. No mutations were detected in non-Hurthle cell carcinomas or blood donor samples. One of the sporadic Hurthle cell papillary carcinomas with a *GRIM-19* mutation also had a *RET/PTC-1* rearrangement. Interestingly, no *GRIM-19* mutations were identified in familial Hurthle cell tumors, suggesting that *GRIM-19* may not be the *TCO* gene. The *GRIM-19* mutations may contribute to the development of sporadic or familial Hurthle cell tumors by affecting *GRIM-19*’s role in mitochondrial metabolism and cell death (37).

### 5.20 19p13.2 *TIMM44*

This particular study (36) focused on 8 families affected by oncocytic thyroid carcinomas. The researchers conducted a systematic screening of 14 candidate genes and observed their localization in regions associated with affected members of the families with oncocytic thyroid carcinomas.

Through this screening, the study identified two new variants in *TIMM44*. These variants were located in exon 9 and exon 13 of the gene, specifically at 19p13.2. *TIMM44* is known as a mitochondrial endosomal transporter involved in the import of nuclear-encoded proteins into mitochondria. Interestingly, these *TIMM44* variants were found to co-occur with the oncocytic thyroid carcinomas phenotype within the affected families.

### 5.21 19p13.2 *MYO1F*

A studies (38) revealed a novel heterozygous mutation in exon 5 of the *MYO1F* gene (c.400G > A, NM: 012335; p.Gly134Ser) that maps to the linkage site. In addition to this mutation, another variant was found in exon 7 among the 192 FNMTC families studied.

This variant leads to an increase in mitochondrial mass and significantly elevates reactive oxygen species levels. Furthermore, it confers a significant advantage in terms of colony formation, invasion, and anchorage-independent growth. Moreover, there is another variant in exon 7 that causes exon skipping, which is predicted to alter the ATP-binding domain in *MYO1F*.

### 5.22 19q13.33 *NOP53*

A study (12) conducted WES of one family and analyzed an additional 44 FNMTC families using Sanger sequencing. In this study, a shared germline variant in *NOP53*, known as Asp31His (rs78530808, with a minor allele frequency of 1.8%), was identified in three non-syndromic FNMTC families.

Functional studies of *NOP53* in thyroid cancer cell lines have demonstrated its oncogenic function. However, considering the relatively high frequency of this variant in the general population, it suggests that *NOP53* may not be a causative gene but rather a low penetrance gene associated with FNMTC.

## 7 Pathway and process enrichment analysis

We performed pathway and process enrichment analysis on the given gene list above using the following ontology source from Metascape (version v3.5.20230501, https://metascape.org/): KEGG Pathway, GO Biological Processes, Reactome Gene Sets, Canonical Pathways, CORUM, WikiPathways, and PANTHER Pathway. All genes in the genome have been used as the enrichment background. Terms with a *p*-value < 0.01, a minimum count of 3, and an enrichment factor > 1.5 are collected and grouped into clusters based on their membership similarities. The most statistically significant term within a cluster is chosen to represent the cluster.

**Figure 2:**
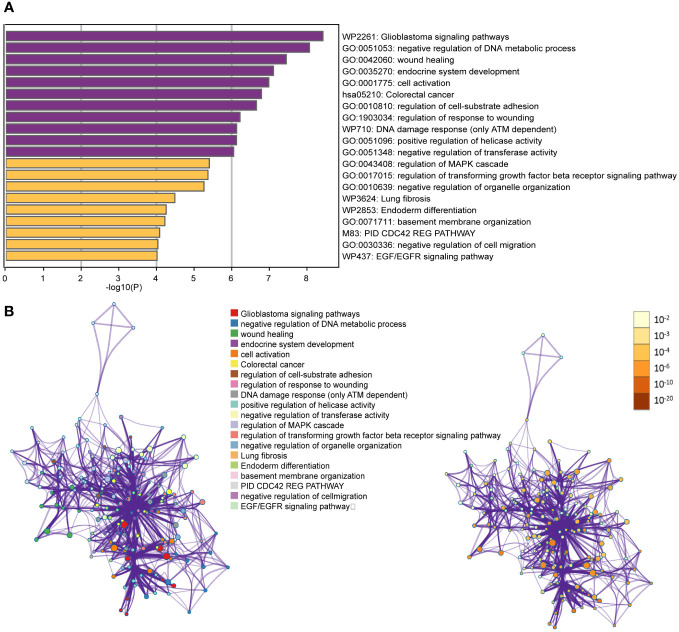
**(A)** Bar graph of enriched terms across input gene lists, colored by p-values; **(B)** Network of enriched terms: colored by cluster ID, where nodes that share the same cluster ID are typically close to each other; colored by p-value, where terms containing more genes tend to have a more significant p-value.

According to **Figure 2A**, we have found that the currently reported genes are mainly involved in key processes related to carcinogenesis, such as negative regulation of DNA metabolic process, endocrine system development, cell activation, and regulation of cell-substrate adhesion in GO Biological Processes. Additionally, key pathways associated with thyroid cancer, such as DNA damage response (only *ATM* dependent) and EGF/EGFR signaling pathway, were identified in WikiPathways.

To enhance the representation of term relationships, we have created a network plot using a subset of enriched terms. In this network, terms with a similarity greater than 0.3 are connected by edges. Our selection process involved choosing terms with the best *p*-values from each of the 20 clusters. We ensured that each cluster had no more than 15 terms and that the total number of terms did not exceed 250. The resulting network is visualized using Cytoscape (111), where each node represents an enriched term. Nodes are initially colored based on their cluster ID and then further differentiated by their p-value (**Figure 2B**).

## 8 Protein-protein interaction enrichment analysis

**Figure 3:**
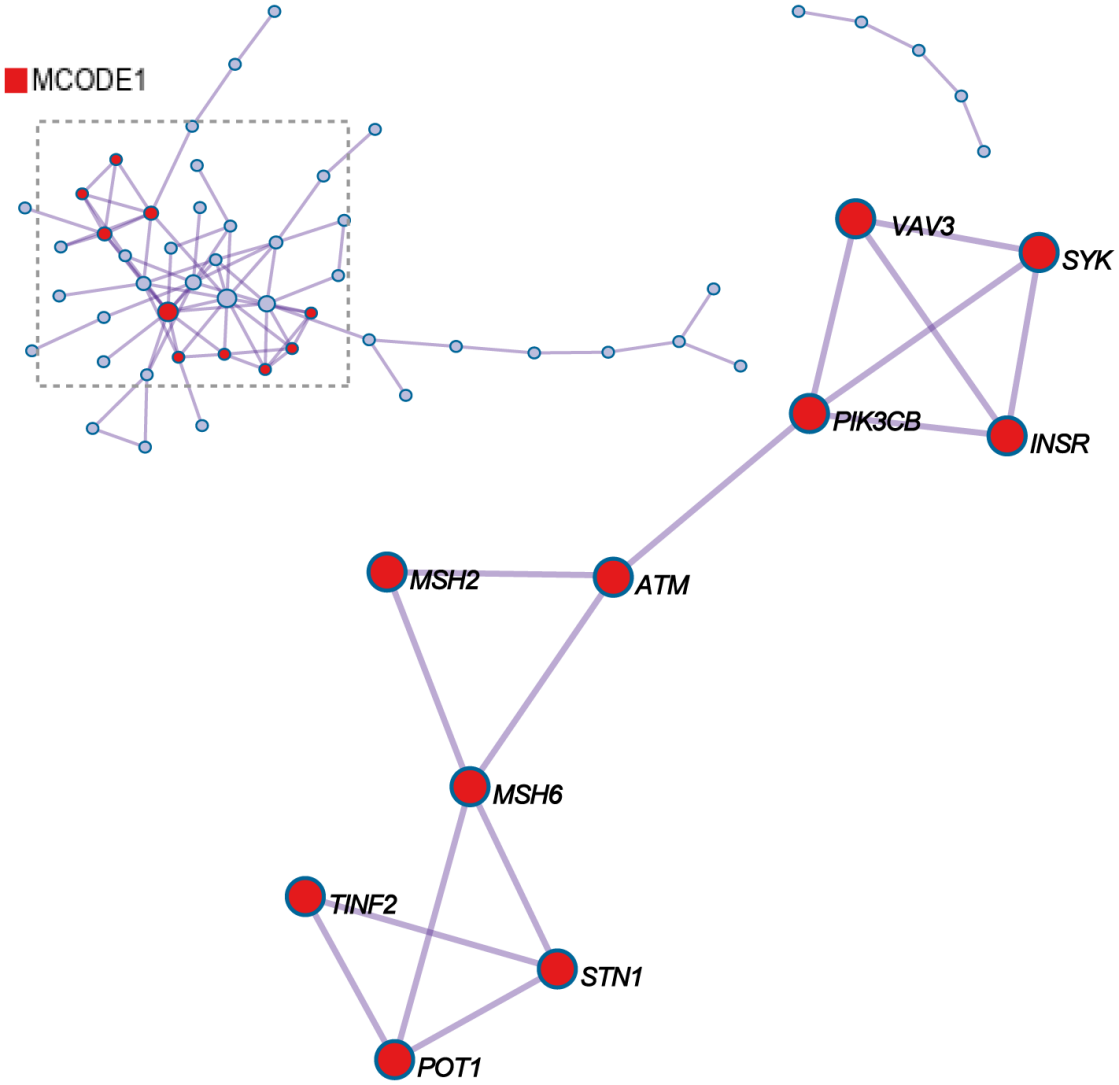
Protein-protein interaction network and MCODE components identified in the gene lists.

For each given gene list, protein-protein interaction enrichment analysis has been carried out with the following databases: STRING6, BioGrid7, OmniPath8, InWeb_IM9.Only physical interactions in STRING (physical score > 0.132) and BioGrid are used. MCODE_1 shows the three best-scoring terms (GO:0051053, negative regulation of DNA metabolic process, Log10(P)= -11.6; GO:0032205, negative regulation of telomere maintenance, Log10(P)= -9.5; GO:0051096, positive regulation of helicase activity, Log10(P)= -9.0) by p-value have been retained as the functional description of the corresponding components, shown in the tables underneath corresponding network plots within **Figure 3**. *VAV3*, *SYK*, *PIK3CB*, *ATM*, *MSH2*, *MSH6*, *TINF2*, *STN1*, and *POT1* potentially have protein-protein interaction relationships among them.

## 9 Discussion

Currently, no significant loci have been successfully identified in the research on FNMTC, indicating that the genetic causal model of FNMTC may be complex and mostly validated only in individual pedigrees, without genetic replication in other pedigrees. This may be related to various factors, with racial differences being one possible reason. Another important factor is the genetic heterogeneity of inherited diseases, where different gene variations can lead to the same disease, or in other words, a single factor does not determine a particular disease.

The strength of this article lies in its first-time summary of the chromosomal landscape map, which facilitates researchers to visually identify gene clustering loci. We also provide a summary of the localization and methodologies of susceptibility genes from different countries. The integration presented in this study may provide new insights for further research.

However, the limitation of this study is that FNMTC susceptibility genes are family-specific pathogenic genes, and each family may have its unique mechanisms of disease development. Currently, there is no precise localization of the causative genes for this condition.

It is indeed worth discussing the potential avenues for future research in the field of FNMTC, such as the development of new assessment methods like PRS and FCVPP. Researchers may uncover novel insights into the molecular mechanisms underlying FNMTC by investigating gene expression patterns, studying individual cells, and examining copy number variations in the future. These investigations could potentially lead to the identification of biomarkers for early detection, as well as the development of targeted therapeutic approaches.

## 10 Conclusion

This review provides an overview of the recent advancements in non-syndrome FNMTC susceptibility gene research, offering comprehensive theoretical support for subsequent investigations in this field. To explore the pathogenesis of FNMTC, more FNMTC pedigrees need to be included to expand and improve the FNMTC gene database. For non-syndromic FNMTC patients, early detection, diagnosis, and treatment may be an effective way to prevent disease progression.

## Author contributions

YJ-J: Conceptualization, Data curation, Formal analysis, Methodology, Writing – original draft. YX: Conceptualization, Data curation, Formal Analysis, Methodology, Writing – original draft. Z-JH: Data curation, Writing – original draft. Y-XH: Writing – original draft, Writing – review & editing. TH: Conceptualization, Methodology, Writing – review & editing.
